# Chemical composition and antibacterial activity of *Berberis vulgaris* (*barberry*) against bacteria associated with caries

**DOI:** 10.1002/cre2.379

**Published:** 2020-12-15

**Authors:** Maryam Kazemipoor, Pooya Fadaei Tehrani, Hengameh Zandi, Reza Golvardi Yazdi

**Affiliations:** ^1^ Department of Endodontics, Faculty of Dentistry Shahid Sadoughi University of Medical Sciences Yazd Iran; ^2^ Dental Students Research Center, Faculty of Dentistry Shahid Sadoughi University of Medical Sciences Yazd Iran; ^3^ Department of Microbiology, Faculty of Medicine Shahid Sadoughi University of Medical Sciences Yazd Iran

**Keywords:** antimicrobial activity, berberine, *Berberis*, dental caries, medicinal plant, natural medicine

## Abstract

**Objectives:**

The aim of this in‐vitro study was to determine the antimicrobial capacity of a *Berberis vulgaris* plant extract on the bacteria being associated with caries including, *Streptococcus mutans*, *S*. *sobrinus*, *S*. *sanguinis*, *S*. *salivaris* and *Lactobacillus rhamnosus*.

**Material and methods:**

Chlorhexidine 2% (CHX) mouthwash and ampicillin (10 μg/disk) were applied as positive control groups. Inhibition zone, minimum inhibitory concentration (MIC) and minimum bactericidal concentration (MBC) related to stem, leaf and fruit of *B*. *vulgaris* plant were recorded for every five bacteria. Data were analyzed using SPSS ver. 22, one‐way ANOVA repeated measure and post hoc Tukey statistical test. The significance level was set at *p* < 0.05.

**Results:**

There were no significant differences between the antimicrobial capacity of the positive controls and the extract from the stem and fruit of *B*. *vulgaris* (*p* > 0.05). The MIC values of the extract from the stem were significantly lower against *S*. *sobrinus* (64 μg/ml) and *L*. *rhamnosus* (128 μg/ml). The MIC value of the extract against *S*. *mutans* was significantly lower in the fruit group (64 μg/mL). The MBC value of the extract against *S*. *sobrinus* and *L*. *rhamnosus* was significantly lower in the stem group (128 μg/ml). The MBC value against *S*. *mutans* was significantly lower in the fruit group (128 μg/ml).

**Conclusions:**

The results showed that CHX and *B*. *vulgaris* plant extract have similar antimicrobial activity against bacteria being associated with caries. Therefore, *B*. *vulgaris*, which shows antibacterial capacity, could be considered for further investigation as a safe, phytotherapeutic mouthwash to prevent dental caries.

## 1 INTRODUCTION

Dental caries is the most common infectious disease in the oral cavity (Mohammadi‐Sichani et al., 2016) and if it is not prevented, it can result in destruction of dental structures followed by pulpitis and periapical lesions. Keyes and Newbrun in 1960s proposed four key elements in formation of dental caries: caries‐causing bacteria, fermentable carbohydrates, susceptible tooth surface and time (Keyes, 1969; Newbrun & Sharma, 1976).

Bacterial biofilm is the main etiological factor inducing dental caries (Allaker & Douglas, 2009). Destruction of dental structure occurred through the fermentation of sucrose and producing lactic acid by cariogenic bacteria (Yadav & Prakash, 2016). *Streptococcus mutans (S*. *mutans)* is the main bacterium of dental biofilm (Loesche, 1986). Lactic acid which produced by *S*. *mutans*, demineralizes hard dental tissues and this process makes the environment more suitable for other bacteria to grow (Westergren & Emilson, 1982).

Although the primary intervention to prevent dental caries is the mechanical removing of oral biofilm (Qiu et al., 2020) but application of antibiotics has also been suggested in a few studies to overcome dental caries (Lima et al., 2005). Antibiotics alone could not inhibit demineralization process occurred in dental biofilm and may lead to antibiotic resistance due to the formation of biofilm in dental biofilm (Kouidhi et al., 2015). Maintenance of ecological balance in microbial community and application of a combination of antimicrobial therapy with different mechanisms plays an important in control caries process (Yeke & Tao, 2012). Antibiotics change oral microbiota and contribute to development of resistant bacteria (Järvinen et al., 1993). Other common side effects of antibiotics including vomiting, diarrhea and discoloration of teeth may also been observed (Chung et al., 2006).

Mouth washes are also recommended to control dental biofilm (Browning et al., 2000). Due to the frequent use of mouthwashes, the introduction of a material with minimal side effects (pigment formation on dental surfaces, infections, and tissue toxicity) and maximum beneficial effects (biofilm control) is essential (Amirzade‐Iranaq & Masoumil, 2017; Wilson et al., 2014). In comparison to synthetic mouthwashes, herbal drugs have more economic efficiency besides fewer side effects (Bagheri et al., 2016).

Barberry (*Berberis vulgaris* L. a family of *Berberidaceae*) grows in Asia and Europe. The *B*. *vulgaris* is a shrub, about 1–3 m tall, with spiny, yellow wood and obviate leaves that have yellow flowers succeeded by oblong red berries (Ciulei et al., 1993; Damaschin, 2006; Dewick, 2002). Various parts of this plant, including the roots, bark, leaves, and fruit, have been used for medicinal purposes. The main alkaloids in this plant are berberine, berbamine and palmatine. Its main components have various therapeutic effects (Fatehi et al., 2005; Imanshahidi & HJ, 2008; Javadzadeh & Fallah, 2012). Its powerful antimicrobial capacity against *Staphylococcus aureus* (*S*. *aureus*) and *Candida* spp. has been shown in the literature (Freile et al., 2003). Other in vitro studies have shown that the berberine is effective against *Entamoeba histolytica*, Giardia Lambia, *Trichomonas vaginalis* (Kaneda et al., 1991), *Helicobacter pylori* (Mahady et al., 2003) and *Leishmania donovani* (Ghosh et al., 1985).

In the range of studies conducted on *B*. *vulgaris*, the effect of this plant on dental caries microorganisms has not been studied and insufficient information is available in this regard. Therefore, the purpose of this study was to investigate the antibacterial effect of Barberry's extract on five common bacteria being associated with caries. Since the antimicrobial capacity of an extract depends on active ingredients, we have applied *Gas chromatography–mass spectrometry (GC–MS) analysis* to determine the chemical composition of the extract derived from the stem, leaf and fruit of *Berberis* plant.

## 2 MATERIALS AND METHODS

### 2.1 Plant material and extraction procedure

This study was approved by Shahid Sadoughi University of Medical Sciences Ethics Committee Yazd, Iran (IR.SSU.REC.1397.107). *B*. *vulgaris* samples were collected by the author from the orchards of Semirom city of Isfahan province and were identified by botanical experts and registered in the herbarium of the Ministry of Jihad Agriculture of Iran with the identification number IRAN 77354. Stem, fruit, and leaf parts of *Berberis* were separately shade, dried and powdered using a blender. Percolation method was used for alcoholic extraction of plant parts. Briefly, a plant powder was mixed with 70% ethyl alcohol at a ratio of 1:5 and stand for 48 h. Afterward, extracts were filtered twice using Whatman Nos. 4 and 1 filter papers (Pharmagona, Manchester, England) and centrifuged. The traces of ethanol and water were removed by keeping the extracts in water bath (40°C) and in presence of calcium chloride, respectively. Extracts from different parts of *Berberis* plant were sterilized by 0.45 μm micro pore filters.

### 2.2 GC–MS analysis

For GC–MS analysis, the ground dried stem, fruit and leaf (2 g) of the plant were soaked in water (50 ml) at room temperature overnight. Afterward, the aqueous solution was evaporated under reduced pressure and the dried extract was dissolved in ethanol and passed through a 0.45 μm filter before injecting into the chromatograph.

The Hewlett‐Packard 5971 GC–MS device (Avondale, PA), available at the Islamic Azad University, Isfahan (Khorasgan) Branch, was applied to determine the chemical composition of the extract derived from the stem, leaf and fruit of *Berberis* plant. The GC–MS device had the following settings: 0.25 mm × 30 m polydimethylsiloxane DB‐1 fused silica capillary column, 0.10‐μm film thickness, 1 ml/minute carrier gas of helium, injector temperature of 250°C, and detector temperature of 200°C. The column temperature was set variable from 35°C/min to 180°C/min at 4°C V/min followed by 180°C/min to 280°C/min at 20°C V/min. The electronic impact of 70 eV was considered for the mass spectra.

### 2.3 Bacterial strains preparation

Standard bacterial strains, including: *S*. *mutans (PTCC 1683)*, *S*. *sobrinus (PTCC 1601)*, *S*. *sanguinis (PTCC 1449)*, *S*. *salivaris (PTCC 1448) and Lactobacillus rhamnosus (PTCC 1637)* were purchased from Iranian Research Organization for Science and Technology (IROST), Tehran, Iran. Bacterial colonies of *S*. *mutans*, *S*. *sobrinus*, *S*. *sanguinis*, *S*. *salivaris* and *L*. *rhamnosus* were obtained after incubation on blood agar medium for 24 h at 37°C.

### 2.4 Screening of antimicrobial activity

The antimicrobial screening was performed using the disk diffusion assay (Andrews, 2001). A turbidity equivalent to 0.5 McFarland turbidity standard, holding 1.5 × 10^8^ colony‐forming units per milliliter (CFU/ml), was adjusted by inoculating colonies of fresh bacterial cultures into 5 ml of sterile broth medium, followed by inoculation of suspensions of Streptococcal strains and *L*. *rhamnosus* on Mueller‐Hinton agar medium enriched with 5% defibrinated sheep blood (MHSBA; Merck, Darmstadt, Germany) and on De Man, Rogosa, and Sharpe (MRS) agar medium (Merck, Darmstadt, Germany), respectively. Sterile blank filters paper disks, 6 mm in diameter (Padtan Teb Co., Iran) were individually impregnated with 100 μl of the extract (10 mg/ml) to give a final concentration of 1 mg/disk and left to dry. Extract disks were then placed on the seeded agar plates and were kept at 4°C for 1 h for diffusion of extract. Plates were incubated at 35°C for 24–48 h in 5% carbon dioxide (CO_2_) condition. After incubation, the diameter (mm) of non‐growth zones around disks was measured in comparison with 2% Chlorhexidine and ampicillin (10 μg/disk) as standards and normal saline as control. The test was repeated three times for each bacterial strain and extract. The inhibition zone was measured in millimeter, and the results recorded as mean ± *SD*. Data were analyzed statistically by ANOVA and Tukey HSD test.

### 2.5 Determination of minimum inhibitory concentration and minimum bactericidal concentration

After confirmation of the antibacterial effect of *B*. *vulgaris* extract from stem, fruit and leaf, broth micro dilution method (Basri & SJIjoP, 2005) according to CLSI 2016 protocols was carried out to determine minimum inhibitory concentration (MIC) values. Plant extracts were suspended into DMSO 10% (which has no activity against test microorganisms) to make 256 μg/ml final concentration. After that two‐fold serially diluted and added to Mueller Hinton broth medium (MRS broth for *L*. *rhamnosus* strain) of 96‐wells of microtiter plates. Hundred microliters bacterial inoculum (1 × 10^8^ CFU/ml) was added to each well. Wells containing bacterial suspensions and broth media with extracts were used as negative and positive control, respectively.

The microtiter plates were incubated at 35°C for 24 h in 5% CO_2_ condition. The well of the microtiter plate that showed no turbidity after incubation was taken as the lowest concentration of the extract (MIC value). The MIC value according to different extracts for each bacterial strain was assayed in triplicate.

The minimum bactericidal concentration (MBC) was determined by inoculating of 50 μl from each well showing no apparent growth on the MHSBA and MRS agar media for streptococcal strains and *L*. *rhamnosus* strain, respectively. Least concentration of each extract showing no visible growth on media was reported for MBC. The data were analyzed using SPSS ver. 22 (SPSS Inc., Chicago, IL), one‐way ANOVA repeated measure and post hoc Tukey statistical test.

## 3 RESULTS

**FIGURE 1:**
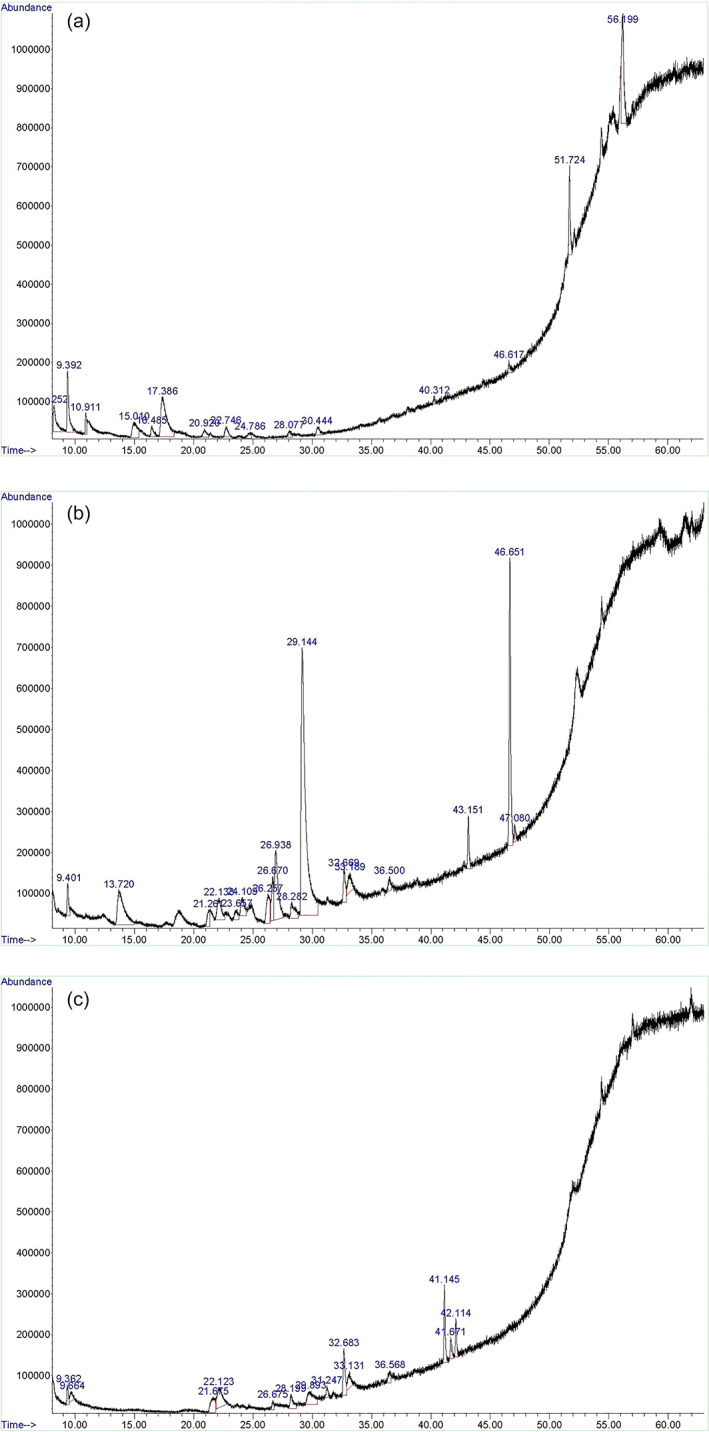
GC–MS chromatogram of the *Berberis vulgaris* alcoholic extract: (a) Stem, (b) Fruit, (c) Leaf

The GC–MS findings (detection at 252 nm) exhibited that the main compounds found in the ethanolic extract from stems, fruit and leaf of *B*. *vulgaris* were 1,1,1,5,7,7,7‐Heptamethyl‐3,3‐bis(trimethylsiloxy)tetrasiloxane (25.2%), 2‐Furaldehyde, 5‐(hydroxymethyl) (51.60%) and phenol (14.47%) respectively (Figure 1).

The results of the antibacterial testing of *B*. *vulgaris* aqueous ethanolic extract from stem, fruit and leaf in comparison with Chlorhexidine and ampicillin are presented in Table 1 (the *p*‐value recorded in Table 1 is only for the 1 mg/disk concentration of the extract).

**TABLE 1:** In vitro comparison of antibacterial activity related to extracts from stem, fruit and leaf of *B*. *vulgaris* against cariogenic bacteria

Bacterial strains	Diameter of zone of inhibition (mm)					
	Stem	Fruit	Leaf	Control		
				CHX	Ampi	*P*‐value^a^
*S*. *sobrinus*	14.0 ± 0.94	7.3 ± 1.36	6.0 ± 3.40	18 ± 0.98	16 ± 0.79	0.010
*S*. *sanguinis*	9.0 ± 1.36	7.6 ± 0.78	6.0 ± 2.33	15 ± 0.80	15 ± 0.86	0.011
*S*. *salivaris*	11.3 ± 1.24	10.0 ± 1.23	9.3 ± 0.98	16 ± 1.23	14 ± 1.34	0.011
*S*. *mutans*	7.6 ± 0.78	8.0 ± 2.23	6.3 ± 1.33	16 ± 0.48	14 ± 1.65	0.010
*Lactobacill rhamnosus*	7.8 ± 1.24	6.0 ± 1.25	6.6 ± 2.64	17 ± 1.24	16 ± 0.68	0.013

*Note*: Data are presented as mean ± *SD*.

Abbreviations: CHX, chlorhexidine; Ampi, ampicillin.

^a^ One‐way ANOVA repeated measure.

Based on Tukey HSD test, in *S*. *sobrinus* bacterium, there was only significant difference between the antibacterial effect of leaf extract and CHX (*p* = 0.020). In *S*. *sanguinis*, *S*. *salivaris* and *S*. *mutans* there were only significant differences between the antibacterial effect of leaf extract and ampicillin respectively (*p* = 0.028, *p* = 0.015, *p* = 0.022). In *L*. *rhamnosus*, there was only significant difference between the antibacterial activity of fruit extract and ampicillin (*p* = 0.020).

Two‐fold serial dilution ranges were between 8 and 256 μg/ml and the MIC value according to different extracts for each bacterial strain was assayed in triplicate. The MIC values listed in Table 2 are the average of the three MICs of each extract for each bacterial strain. The MIC values of the stem extract were significantly lower against *S*. *sobrinus* and *L*. *rhamnosus*. The MIC values of the fruit extract were significantly lower against *S*. *sanguinis* and *S*. *mutans* (Table 2).

**TABLE 2:** MIC values of *B*. *vulgaris* extracts against cariogenic bacteria

Bacterial strains	MIC (μg/ml)			*P*‐value^a^
	Stem	Fruit	Leaf	
*S*. *sobrinus*	64	128	>256	0.018
*S*. *sanguinis*	85.33 ± 36.95	64	>256	0.030
*S*. *salivaris*	64	64	64	1
*S*. *mutans*	170.66 ± 73.90	64	>256	0.040
*Lactobacill rhamnosus*	128	>256	>256	0.029

*Note*: Data are presented as mean ± *SD*.

^a^ One‐way ANOVA repeated measure.

Based on post hoc Tukey statistical test, in *S*. *sobrinus*, *S*. *sanguinis* and *L*. *rhamnosus* there were significant differences between the MIC values of stem and leaf extract respectively (*p* = 0.014, *p* = 0.043, *p* = 0.026). In *S*. *mutans* there was significant difference between the MIC values of fruit and leaf extract.

The MBC values attributed to bacterial strains and *B*. *vulgaris* extracts are summarized in Table 3. The MBC value of fruit extract was significantly lower against *S*. *mutans* (*p* = 0.044). In contrast, the MBC values of stem extract were significantly lower against *S*. *sobrinus* and *L*. *rhamnosus* respectively (*p* = 0.0.018, *p* = 0.029).

**TABLE 3:** MBC values of *B*. *vulgaris* extracts against cariogenic bacteria

Bacterial strains	MBC (μg/ml)			*P*‐value^a^
	Stem	Fruit	Leaf	
*S*. *sobrinus*	128	256	>256	0.018
*S*. *sanguinis*	128	128	>256	0.018
*S*. *salivaris*	128	128	128	1
*S*. *mutans*	256	128	256	0.044
*Lactobacill rhamnosus*	128	>256	>256	0.029

^a^ One‐way ANOVA repeated measure.

## 4 DISCUSSION

Dental caries is a multifactorial disease and bacteria play an important role in incidence and progression of this disease (Nishikawara et al., 2007). Clarke in 1927 isolated streptococci from human carious lesions and named them *S*. *mutans* and future studies revealed that *Mutans streptococci* (MS) and *lactobacilli* bacterium are involved in the development of dental caries (Clarke, 1924; Nishikawara et al., 2007).

Among the seven subtypes of the MS microbiota, the two species of *S*. *mutans* and *S*. *sobrinus* are mostly isolated from dental biofilm (Hirasawa & Takada, 2002). Therefore, the two types *S*. *sobrinus* and *S*. *mutans* have been applied as an important indicator for evaluation of caries risk (Loesche, 1986; Yadav & Prakash, 2016).

Preventive approaches on the incidence and progression of caries lesion reduces the risk of tooth damage and tooth loss during the human life (Yadav & Prakash, 2016). Considering *S*. *mutans*, *S*. *sobrinus* and *lactobacilli* as the main bacteria that involved in the formation of caries lesion, antibacterial regimen should mostly focus on these species. In regard to adverse effects of synthetic antibiotics to prevent dental caries, herbal substitutes have been recommended in the form of mouthwashes as new therapeutic agents in preventive dentistry (Chung et al., 2006; Mohammadi‐Sichani et al., 2016). To the best of our knowledge, there have been no reports on the antibacterial effect of stem, leaf and fruit extracts of *B*. *vulgaris*. The aim of this study was to investigate the antimicrobial capacity of a *B*. *vulgaris* plant extract on the oral pathogens associated with caries including, *S*. *mutans*, *S*. *sobrinus*, *S*. *sanguinis*, *S*. *salivaris* and *L*. *rhamnosus*.

Barberry (*B*. *vulgaris*) is from the family of *Berberidaceae* that has been used extensively in traditional medicine. Pharmacologic studies have shown various therapeutic effects such as vasorelaxant and hypotensive (Fatehi‐Hassanabad et al., 2005), immunomodulation and anti‐inflammatory (Javadzadeh & Fallah, 2012), central nervous system (Peng et al., 2004), endocrine (Lou et al., 2011), respiratory (Lee et al., 2003), gastrointestinal (Lin et al., 2005), skin (Seki & Morohashi, 1993) and antimicrobial effects (Zarei et al., 2015). Berberine, berberamine and palmatine are the main alkaloid constituents that have been detected in this plant. Also, other alkaloids have some roles in the therapeutic effects of this plant extract (Javadzadeh & Fallah, 2012).

Based on the results of the present study, the *Berberis* extract from the stem part of the plant showed the highest antimicrobial effect against *S*. *sobrinus*, *S*. *sanguinis*, *S*. *salivaris* and *L*. *rhamnosus* in comparison to fruit and leaf extract. In the present study, the Barberry stem extract showed antimicrobial efficacy against bacteria being associated with caries, comparable to CHX (a widely used mouthwash) and ampicillin as positive controls.

Berberine (the main alkaloid in the Berberis extract) showed a significant antibacterial and antifungal activity against *Staphylococcus aureus* (*S*. *aureus*) and different *Candida* spp. (Freile et al., 2003). Antibacterial capacity of this extract against oral pathogens being associated with caries has not been studied up to the present (Imanshahidi & HJ, 2008). This extract has an inhibitory effect on the two virulence factors of sortase A (SrtA) and B (Srt B), that are mainly found in *S*. *aureus* (Oh et al., 2006). Sortase A enzyme that also produced with the *S*. *mutans species* is responsible for host cell attachment and biofilm formation (Wang et al., 2019). Berberine also interfere with the process of multi‐drug resistant efflux pumps (mostly observed in *S*. *aureus*) and prevents the adherence of Group A streptococci to host cell (Stermitz et al., 2001).

In the present study, the MIC values of the stem extract against *S*. *sobrinus* and *L*. *rhamnosus* were lower in comparison to fruit and leaf extract. In contrast, the MIC value of the fruit extract was lower against *S*. *mutans*. With regard to MBC records, the stem extract showed better results against *S*. *sobrinus* and *L*. *rhamnosus*, while against *S*. *mutans*, the MBC value was lower in the fruit extract group.

The extract concentration for antibacterial assay in the present study was determined as 10 mg/ml. Freile et al. (2003) in a study evaluated the antimicrobial activity of aqueous extracts of stem, root and leaf of Berberis and pure berberine against *S*. *aureus*, *Enterococcus faecalis* and different *Candida* spp. In the two extract concentrations of 500 and 1000 μg/ml that were applied in the Freile et al. (2003) study, no antibacterial activity was observed against the aforementioned bacteria. In contrast, pure berberine in concentrations of 50, 100 and 200 μg/ml showed high antibacterial activity against *S*. *aureus* and *Candida albicans*. Since in the present study the extracts concentration was higher (10 vs. 500–1000 μg/ml), antibacterial activities were observed in all the experimental groups. It has been shown that the therapeutic dosage of *B*. *vulgaris* for most clinical situation is 200 mg orally, two or four times daily (Birdsall, 1997). Therefore, increasing the extract concentration below the therapeutic dosage, may lead to higher antibacterial capacity and no side effects. Similar to the results of the present study, the MIC values of pure berberine against different *Candida* spp. in the Freile et al. (2003) study, ranged from 64 to 128 μg/ml.

In the present study, GC–MS analysis of the *Berberis* stem extract revealed that the predominant bioactive compounds in the stem extract of this medicinal plant were Heptamethyl tetrasiloxane (25.2%). Based on the results of Hosseinihashemi et al. (2015) study, the major components identified in extracts from the inner bark of *B*. *vulgaris* stem were tetracosanoic acid, methyl ester (26.36%), followed by phthalic acid, diisooctyl ester (20.93%), 1,2‐bis(trimethylsiloxy) ethane (10.26%), and 1,2‐benzendicarboxylic acid, diisononyl ester (8.70%). According to NAPALERT, the main isoquinoline alkaloid found in the stem was Berberine and in a quantitative HPLC analysis, the main alkaloids in the roots, barks and stems of *B*. *vulgaris* were recorded as 1.24% berberine and 2.5% berbamine (Imanshahidi & HJ, 2008). In the present study, berberine, the pre‐dominant bioactive compound, detected only in the stem and fruit extracts, with concentrations of 3.05% and 2.97% respectively. Considering the antimicrobial capacity of different parts of the plant, the leaf extract without detection of any active ingredient in GC–MS analysis, showed the least antibacterial capacity. In contrast the stem extract showed the highest antibacterial effect against the examined bacteria except *S*. *mutans*. Since the active ingredients of the extracts, excluding berberine was different in stem and fruit parts of the plant, different antibacterial effect has been observed.

Several methodologies have been introduced to evaluate the antimicrobial capacity of plant constituents. Disk diffusion method is a relatively insensitive and semi‐quantitative technique that based on diffusion of an antibacterial agent through agar during time (Balouiri et al., 2016). The filter blank papers utilized in this study had a good absorbability for the Berberis extract and could be applied to assess the inhibition of growth in the bacteria being associated with caries.

Environment is an important factor which can affect the phytochemical profile and antibacterial capacity of a plant at a given time. Thus, geographic region of plant growth, the season of plant collection, climate, and plant storage conditions may influence the chemical composition and biological activity of a medicinal plant (Tabrizizadeh et al., 2018). Therefore, to avoid diverse values regarding a medicinal plant antibacterial effect, extract standardization is necessary.

The chemical and biological reactions of *barberry*'*s* extract in the oral cavity require time to exert their effects on cell viability, and this will be the subject of a future study in order to standardize the preparation of barberry's extract as mouthwash. The intention is also to examine and compare the cytotoxicity and antimicrobial efficacy of the individual major metabolites present in the barberry's extract against important cariogenic bacteria in comparison to other interventions like fluoride therapy.

This novel approach investigated the antimicrobial activity of Berberis extracts in comparison to synthetic drugs. Nevertheless, for clinical application of this plant extract in preventive dentistry, more comprehensive studies are required to address the antibacterial activity in the clinical situation and effective concentrations of this medicinal plant against bacteria being associated with caries.

## 5 CONCLUSION

The present study revealed that extracts obtained from stem and fruit of *B*. *vulgaris* show antimicrobial effects against cariogenic oral pathogens. These findings suggest that for clinical application of *B*. *vulgaris* as a safe, anticariogenic, and phytotherapeutic mouthwash, further investigations could be considered.

## CONFLICT OF INTEREST

The authors declare that there are no conflicts of interest regarding the publication of this article.

## AUTHOR CONTRIBUTIONS

All authors have contributed to the research and writing of the article.

## CLINICAL SIGNIFICANCE

This in vitro study was evaluated the antimicrobial capacity of a *Berberis vulgaris* plant extract on the cariogenic oral pathogens that involves in dental caries. We cultured the microorganisms and applied barberry extract to them. Chlorhexidine and amoxicillin disks were used as controls. The present study revealed that extracts obtained from stem and fruit of *B*. *vulgaris* show antimicrobial effects against cariogenic oral pathogens. These findings suggest that for clinical application of *Berberis vulgaris* as a safe, anticariogenic, and phytotherapeutic mouthwash, further investigations could be considered.

## Data Availability

All data generated or analysed during this study are included in this article.
